# Phase II study of the c-MET inhibitor tivantinib (ARQ 197) in patients with relapsed or relapsed/refractory multiple myeloma

**DOI:** 10.1007/s00277-017-2980-3

**Published:** 2017-03-23

**Authors:** Muhamed Baljevic, Shadia Zaman, Veerabhadran Baladandayuthapani, Yan Heather Lin, Claudia Morales de Partovi, Zuzana Berkova, Behrang Amini, Sheeba K. Thomas, Jatin J. Shah, Donna M. Weber, Min Fu, Charles S. Cleeland, Xin Shelley Wang, Christine M. Stellrecht, Richard E. Davis, Varsha Gandhi, Robert Z. Orlowski

**Affiliations:** 1 Division of Hematology & Oncology, The University of Nebraska Medical Center, Omaha, NE USA; 2 Division of Cancer Medicine, The University of Texas MD Anderson Cancer Center, Houston, TX USA; 3 Department of Experimental Therapeutics, The University of Texas MD Anderson Cancer Center, Houston, TX USA; 4 Department of Biostatistics, The University of Texas MD Anderson Cancer Center, Houston, TX USA; 5 Department of Lymphoma/Myeloma, The University of Texas MD Anderson Cancer Center, Houston, TX USA; 6 Department of Diagnostic Radiology, The University of Texas MD Anderson Cancer Center, Houston, TX USA; 7 Department of Symptom Research, The University of Texas MD Anderson Cancer Center, Houston, TX USA

**Keywords:** ARQ 197, Tivantinib, c-MET, Multiple myeloma, Relapsed

## Abstract

The hepatocyte growth factor/c-MET pathway has been implicated in the pathobiology of multiple myeloma, and c-MET inhibitors induce myeloma cell apoptosis, suggesting that they could be useful clinically. We conducted a phase II study with the c-MET inhibitor tivantinib in patients with relapsed, or relapsed and refractory myeloma whose disease had progressed after one to four prior therapies. Tivantinib, 360 mg orally per dose, was administered twice daily continuously over a 4-week treatment cycle without a cap on the number of allowed cycles, barring undue toxicities or disease progression. Primary objectives were to determine the overall response rate and the toxicities of tivantinib in this patient population. Sixteen patients were enrolled in a two-stage design. Notable grade 3 and 4 hematological adverse events were limited to neutropenia in five and four patients, respectively. Nonhematological adverse events of grade 3 or higher included hypertension (in four patients); syncope, infection, and pain (two each); and fatigue, cough, and pulmonary embolism (one each). Four of 11 evaluable patients (36%) had stable disease as their best response, while the remainder showed disease progression. Overall, tivantinib as a single agent did not show promise for unselected relapsed/refractory myeloma patients. However, the ability to achieve stable disease does suggest that combination regimens incorporating targeted inhibitors in patients with c-MET pathway activation could be of interest.

**Electronic supplementary material:**

The online version of this article (doi:10.1007/s00277-017-2980-3) contains supplementary material, which is available to authorized users.

## Introduction

Multiple myeloma is the second most commonly diagnosed hematologic malignancy, and the total number of new cases may show a 60% increase between 2010 and 2030 [1], indicating that it is an increasing health care burden. Introduction over the past decade of proteasome inhibitors, immunomodulatory agents, and their combinations has improved the outcome of patients with relapsed and relapsed/refractory myeloma [2]. Nevertheless, patients still experience multiple relapses and decreasing remission durations with each additional line of therapy [2], and those with refractory disease in particular may not respond favorably to standard agents. Their use is limited by treatment-emergent toxicity, or by intrinsic or acquired resistance, underscoring the continued need for novel treatments.

Activation of the c-MET receptor tyrosine kinase induced by its ligand, hepatocyte growth factor (HGF), plays a role in myeloma pathobiology. Malignant plasma cells secrete HGF activator (HGFA), which converts HGF to its active form, and high HGF levels correlate with a poor prognosis [3, 4]. Syndecan 1 (CD138) on myeloma cells binds HGF and promotes c-MET signaling [5], while both potentiate interleukin-6-induced growth and migration, correlating with shorter survival [6]. Preclinical studies showed that suppression of c-MET signaling with small molecule inhibitors, including tivantinib, inhibited proliferation and induced apoptosis [7–10]. Preclinically, tivantinib-mediated cytotoxicity was observed at concentrations <5 μM, a level achievable in the clinic that showed activity in solid tumors with a dose of 360 mg twice daily [11]. Phase II single-agent tivantinib studies demonstrated efficacy in solid tumors [12], and the combination of tivantinib and erlotinib was tested in a phase III trial in nonsmall cell lung carcinoma and showed an improvement for the two-drug regimen in progression-free, though not overall survival [13]. Finally, biomarker studies demonstrated the efficacy of tivantinib in inhibiting MET receptor kinase, as well as inducing apoptosis of target tumor cells [11]. Together, these findings supported the hypothesis that suppressing HGF/c-MET signaling could be a rational strategy against relapsed/refractory myeloma, and we therefore performed a phase II clinical trial testing this possibility.

## Patients and methods

### Patient selection

Patients with histologically confirmed symptomatic myeloma by the International Myeloma Working Group (IMWG) criteria with 1–4 prior lines of therapy were eligible. They had to have measurable disease that had relapsed after, or was refractory to the prior regimen. Additional key inclusion criteria included: (1) Eastern Cooperative Oncology Group (ECOG) performance status of 0–2, (2) adequate bone marrow reserves [absolute neutrophil count (ANC) ≥1000 cells/mm^3^, hemoglobin ≥8 g/dL; platelets ≥100,000 cells/mm^3^], and (3) adequate hepatic [total bilirubin ≤1.5× normal, aspartate and alanine aminotransferase ≤2.5× normal], renal [normal serum creatinine, or creatinine clearance ≥30 mL/min], and cardiac function [absence of New York Heart Association class II-IV congestive heart failure, uncontrolled angina, hypertension, myocardial infarction within 6 months, or grade 3–4 cardiac arrhythmia by NCI Common Terminology Criteria for Adverse Events (CTCAE), version 4.0]. Key exclusion criteria included: (1) central nervous system involvement, (2) previous treatment with another agent targeting HGF/c-MET, and (3) nonsecretory myeloma, active plasma cell leukemia, or POEMS syndrome (polyneuropathy, organomegaly, endocrinopathy, monoclonal gammopathy, and skin changes). The MD Anderson Institutional Review Board approved the trial. Informed consent was obtained from all patients for being included in the study. All procedures followed were in accordance with the ethical standards of the responsible committee on human experimentation (institutional and national) and with the Helsinki Declaration of 1975, as revised in 2008. This study was registered with Clinicaltrials.gov (NCT01447914).

### Study design

This open-label, phase II study used a Simon’s Minimax 2-stage design. A response rate of ≤10% was considered as clinically insignificant, while 30% was considered significant. When the probabilities of accepting a “bad” drug and of rejecting a “good” drug are both 0.1, the Simon Minimax 2-stage design required 16 patients in the first stage. Another nine would have been enrolled had there been evidence of ≥1 documented partial remission (PR) or better.

The primary objectives were to determine the overall response rate (ORR) to tivantinib and to define its toxicities. Secondary objectives were (1) to obtain preliminary evidence of response durability, (2) to correlate HGF/c-MET pathway activation in primary cells at baseline with efficacy, and (3) to correlate serum and marrow HGF, HGFA, and soluble c-MET (sc-MET) levels with HGF/c-MET pathway activation in primary cells at baseline and after treatment.

An exploratory objective was to evaluate the symptom burden of patients using the MD Anderson Symptom Inventory (MDASI) and its myeloma module (MDASI-MM). This brief, validated [14], patient-reported outcome tool measured 13 cancer-related symptoms and their impact on daily living. The symptoms assessed included pain, fatigue, nausea, disturbed sleep, distress, shortness of breath, difficulty remembering, lack of appetite, drowsiness, dry mouth, sadness, vomiting, numbness, constipation, muscle weakness, diarrhea, mouth or throat sores, rash, and trouble concentrating. Six additional items assessed symptom-related interference in general activity, mood, work, relation with others, enjoyment of life, and walking. MDASI scores were scored numerically from 0 to 10 (symptom absence to as bad as imaginable). Another exploratory objective evaluated patient-reported outcomes using the European Organization for Research on the Treatment of Cancer (EORTC) Quality of Life Questionnaire (QLQ) Core 30 (QLQ-C30) and the myeloma-specific module QLQ-MY20. Questions relating to overall health and quality of life in the QLQ-C30 were rated from 1 to 7 (very poor to excellent), while the remaining questions were graded qualitatively, in ascending severity, with not at all, a little, quite a bit, or very much.

### Drug dosing and assessments

Tivantinib, provided through the National Cancer Institute Cancer Therapy Evaluation Program (CTEP), was administered at 360 mg p.o. (three tablets of 120 mg) with meals twice daily of every 4-week cycle, and at this dose level, steady-state plasma tivantinib levels in a phase I trial were 7 μM [11]. Treatment was to be continued unless patients experienced undue toxicities, disease progression, or withdrew consent. In the event of treatment-emergent grade 2 rash or hand-foot syndrome, grade 3 nonhematologic toxicities, or grade 4 hematologic adverse events (AEs), the drug was held until these returned to grade 0–1 or the pretreatment baseline. At that point, tivantinib was restarted at a reduced dose (level-1 dose, 240 mg twice daily; level-2 dose, 120 mg twice daily).

### Response and toxicity assessment

An evaluation of disease response by IMWG criteria to determine eligibility to continue therapy was to be performed after the first 2 cycles, and on day 1 of every cycle afterward. Toxicity was monitored throughout treatment, with a special focus on AEs during cycle 1. The toxicity endpoint was defined as any treatment-related unmanageable toxicity, including grade 3 nonhematologic or grade 4 hematologic effects that required delay or treatment termination during cycle 1. Rates of these toxicities ≥25% were considered unacceptable.

### Sample collection

Pretreatment bone marrow samples were sorted into CD138^+^ and CD138^−^ fractions at the MD Anderson Myeloma Tissue Core Facility as described previously [15], and extracted and labeled for gene expression profiling (GEP) [16]. Plasma samples were collected on day 1 of each cycle, and isolated and stored until further analysis. HGF was determined using the HGF enzyme-linked immunosorbent assay (ELISA) (Invitrogen, Camarillo, CA) and analyzed using a Bio-Tek PowerWave XS spectrophotometer (Winooski, VT).

## Results

### Patient characteristics

Sixteen patients enrolled between January, 2012, and April, 2013, and received a median of 3 cycles of therapy, with one patient continuing until February, 2014. All patients were evaluable for toxicity, while 11 were formally evaluable for response after completing 2 cycles. The median patient age was 66 years, and they had received a median of 1 prior line of therapy (range 1–3). Additional demographic data are summarized in Table 1.

**Table 1:** Baseline demographic and clinical characteristics of patients enrolled in this phase II study of tivantinib (*N* = 16)

Characteristic	No.	%
Age (median, range)	66.3 (49–76)	
≤65 years	6	37.5
>65 years	10	62.5
Sex		
Male	9	56.25
Female	7	43.75
Race		
African-American	2	12.5
Asian	1	6.25
Caucasian	13	81.25
Ethnicity		
Hispanic	3	18.75
Non-Hispanic	13	81.25
Disease status		
Relapsed	13	81.25
Refractory	3	18.75
Prior autologous stem cell transplantation		
Yes	10	62.5
No	6	37.5
Prior lines of therapy (median, range)	2.5 (2–3)	
Types of prior therapies		
Bortezomib/lenalidomide/dexamethasone	6	37.5
Cyclophosphamide/bortezomib/dexamethasone	4	25.0
Vincristine/doxorubicin/dexamethasone	1	6.25
Bortezomib/lenalidomide	1	6.25
Bortezomib/Liposomal doxorubicin	1	6.25
Bortezomib/dexamethasone	5	31.25
Thalidomide/dexamethasone	3	18.75
Lenalidomide/dexamethasone	5	31.25
Bortezomib	2	12.5
Lenalidomide	3	18.75
Dexamethasone	1	6.25

### Adverse events and treatment efficacy

There were no unexpected toxicities given the known AE profile of tivantinib and no deaths. The most common AEs in ≥25% of patients, and felt to be at least possibly drug-related, included fatigue or decreased neutrophils (94% each), pain (81%), myalgias (56%), diarrhea (38%), memory impairment, respiratory disorders, and rash (31% each), hypertension (25%), and these were predominantly grades 1–2 (Table 2). Grade 3 or 4 AEs included neutropenia (31 and 25%, respectively), syncope, infection, pain (13% of each, all grade 3), and anal fissure, cough, fatigue, hypertension, and pulmonary embolism (6% each, all grade 3).

**Table 2:** Hematologic and nonhematologic toxicities in patients treated with tivantinib (*N* = 16)

Toxicity grade	1		2		3		4	
	No.	%	No.	%	No.	%	No.	%
Hematological toxicity type								
Neutropenia			15	93.8	5	31.3	4	25.0
Anemia			3	18.8				
Thrombocytopenia^a^	2	12.5						
Nonhematological toxicity type								
Hypertension					4	25.0		
Pain			13	81.3	2	12.5		
Syncope					2	12.5		
Infection					2	12.5		
Fatigue	15	93.8			1	6.3		
Cough^a^	7	43.8			1	6.3		
Pulmonary embolus(i)					1	6.3		
Sinus bradycardia			3	18.8				
Allergic rhinitis^a^			2	12.5				
Insomnia^a^			2	12.5				
Metabolism/ND^a^			2	12.5				
Musculoskeletal/connective TD			2	12.5				
Peripheral sensory neuropathy^a^	10	62.5						
Myalgia	9	56.3						
Skin/subcutaneous TD^a^	8	50.0						
Diarrhea	6	37.5						
Constipation^a^	5	31.3						
Renal/urinary disorder^a^	5	31.3						
Respiratory disorder	5	31.3						
Maculopapular rash	5	31.3						
Memory impairment	5	31.3						
Blurred vision^a^	5	31.3						
Dry eye^a^	4	25.0						
Limb edema^a^	3	18.8						
Fever	2	12.5						
Headache	2	12.5						
Dizziness^a^	2	12.5						
Gastrointestinal disorder^a^	2	12.5						
Nausea	2	12.5						
Watery eyes	2	12.5						
Alopecia	2	12.5						
Dry skin^a^	2	12.5						
Anxiety^a^	2	12.5						

*ND* nutritional deficiency, *TD* tissue disorder

^a^Unrelated to the treatment

Stable disease (SD) was observed as the best response in 4/11 (36%) evaluable patients, or 4/16 (25%) patients who were enrolled and received at least one dose of drug. This was maintained for up to 15 cycles in patient 12, who withdrew consent because other therapies were available, while the remaining 7/11 (63%) patients showed progression (PD). Among the five inevaluable patients, treatment was stopped prior to completing 2 cycles because of toxicity in two patients (syncope/bradycardia and neutropenic fever), withdrawal of consent in one, and PD in two patients (both showing a progressing Bence-Jones paraprotein). While the protocol did allow replacement of patients who were inevaluable for response, after consultation with CTEP, and given the lack of activity, a decision was made to halt enrollment.

**Fig. 1:**
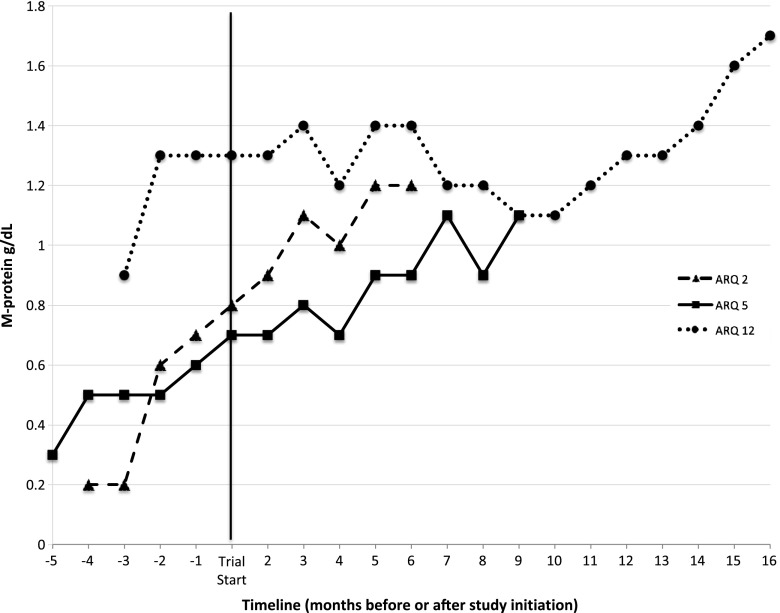
Monoclonal protein evolution prior to and during tivantinib therapy for stable disease patients. Only patients 2, 5, and 12, who experienced longer durations of therapy on protocol, are depicted

Prior therapies received by the patients who experienced SD included thalidomide and dexamethasone (Td) leading to autologous stem cell transplantation (ASCT), and then bortezomib and dexamethasone (Vd) at first relapse in patient 2; Vd, followed by bortezomib and pegylated liposomal doxorubicin after first progression in patient 4; Td and ASCT in patient 5; and Vd, followed after progression by lenalidomide, bortezomib, and dexamethasone and then ASCT in patient 12. The monoclonal protein (M-protein) evolution prior to, and after, initiation of tivantinib therapy in SD patients who received more than 2 cycles of therapy is depicted in Fig. 1. All three patients had rising M-proteins at enrollment (patient 12 also had progressing Bence-Jones paraprotein, while patient 5 had progressing serum free light chains and worsening thrombocytopenia) and met criteria for SD, though further exposure to tivantinib yielded evidence of some benefit only in patient 12. In patients with SD, the median durability of response was 7 cycles (range 2–15) or 6.5 months (range 2–15). Two SD patients ultimately withdrew consent (patients 2 and 5), while patient 4 developed declining performance status that led to discontinuation of treatment.

### Symptom burden and quality of life

A total of 95 MDASI and 93 EORTC measurements were collected, 63% of which were within 3 cycles of therapy. The top five most severe MDASI symptoms were fatigue, pain, numbness, limited activity, and muscle weakness (Table 3). During the first cycle, five patients were asymptomatic (scoring 0–3), and four of these completed at least 3 cycles of therapy and remained asymptomatic. Two patients reported >10 symptoms as moderate/severe (scoring 4–10) that significantly interfered with their activity and enjoyment of life; most of those symptoms did not improve over time. Most commonly reported EORTC symptoms during cycle 1 related to tiredness, feeling less attractive, thinking about illness, weakness, and pain interference with daily activities, while the overall top five EORTC concerns were problems with strenuous activities, tiredness, pain/aches, weakness, and problem with long walks (Table 4). The QLQ-C30 questions on overall health and quality of life were scored high consistently during the treatment despite certain patients experiencing PD. Interestingly, both parameters decreased in two patients with SD.

**Table 3:** The most common reported MD Anderson Symptom Inventory (MDASI) symptoms and their severity during tivantinib treatment

Trial	MDASI Symptom Score, median (range)				
Patient	Pain	Fatigue	Numbness	Muscle weakness	Limited activity
ARQ 1	0 (0)	2 (0–2)	0 (0)	0 (0–2)	0 (0)
ARQ 2^a^	2 (0–2)	3 (0–7)	1 (1–3)	0 (0–2)	0 (0–1)
ARQ 3	6 (2–6)	5 (3–7)	7 (4–8)	5 (3–8)	6 (4–8)
ARQ 4^a^	0 (0)	0 (0)	0 (0–7)	0 (0)	0 (0)
ARQ 5^a^	4.5 (1–6)	3 (1–6)	0 (0)	0 (0)	0 (0–1)
ARQ 6	6 (5–7)	8 (6–9)	7 (6–8)	4 (4–6)	5 (4–7)
ARQ 7	0 (0)	0 (0)	2 (0–2)	0 (0)	0 (0)
ARQ 9	0 (0)	0 (0)	0 (0)	0 (0)	0 (0)
ARQ 12^a^	1 (0–1)	0 (0)	0 (0)	0 (0)	0 (0)
ARQ 13	1 (1–2)	1 (1–2)	0 (0)	0 (0–1)	1 (1)
ARQ 14	0 (0)	2 (1–2)	0 (0)	0 (0)	0.5 (0–2)

^a^Denotes four patients who had stable disease response

**Table 4:** The most commonly reported EORTC QLQ-C30 (multiple myeloma) symptoms and their severity during tivantinib treatment

Trial patient	EORTC QLQ-C30 Multiple Myeloma Symptom Score						
	Qualitative measures (Not at all = 1^a^; A little = 2^a^; Quite a bit = 3^a^; Very much = 4^a^), median (range)					Quantitative measures [1–7], median (range)	
	Trouble dong strenuous activities	Long walk problem	Pain/aches	Weakness	Tiredness	Overall health	Overall quality of life
ARQ 1	1 (1)	1 (1–2)	1 (1)	1 (1)	1 (1–2)	7 (6–7)	7 (6–7)
ARQ 2^b^	2 (1–2)	2 (1–3)	1 (1)	2 (1–3)	1 (1–3)	4 (4–6)	5 (3–6)
ARQ 3	3 (2–4)	2 (1–3)	3 (2–3)	3 (2–3)	3 (2–3)	4 (3–5)	4 (2–5)
ARQ 4^b^	1 (1–2)	1 (1)	1 (1)	1 (1)	1 (1–3)	7 (6–7)	7 (6–7)
ARQ 5^b^	1 (1)	1 (1–2)	1 (1–3)	1 (1)	2 (1–2)	6 (4–6)	6 (5–7)
ARQ 6	3 (3)	3 (3)	2 (2–3)	3 (3–4)	3 (3–4)	5 (2–6)	6 (3–6)
ARQ 7	1 (1)	1 (1)	1 (1)	1 (1)	1 (1)	7 (7)	7 (7)
ARQ 9	1 (1–4)	1 (1)	1 (1)	1 (1)	1 (1)	7 (7)	7 (7)
ARQ 12^b^	1 (1–2)	1 (1)	1 (1–2)	1 (1)	1 (1–2)	7 (2–7)	7 (1–7)
ARQ 13	3 (3)	3 (3)	2 (2)	1 (1)	2 (2)	6 (6)	7 (7)
ARQ 14	1 (1)	1 (1)	1 (1)	1 (1)	2 (1–2)	7 (6–7)	7 (7)

*EORTC QLQ-C30* European Organization for Research and Treatment of Cancer Quality of Life Questionnaire C30

^a^Qualitative measures assigned numbers to allow quantification of symptoms

^b^Denotes four patients who had stable disease response

### Plasma HGF levels

**Fig. 2:**
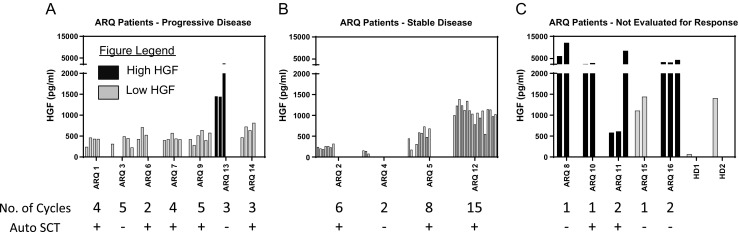
Plasma HGF levels in evaluable patients. Plasma HGF levels (pg/mL) were measured by ELISA in patients with **a** progressive disease, **b** stable disease, and **c** patients who were not evaluable for response. The number of cycles of tivantinib received, and whether the patient had prior autologous stem cell transplant (Auto SCT), is indicated. High HGF samples are labeled with a *black bar*, and low HGF samples are labeled with a *gray bar*. *HD* indicates healthy donors used as controls

A total of 79 plasma samples were collected, and two samples were collected from healthy donors. The median plasma HGF level in myeloma patients was 450 pg/mL (range 150–5991) (Fig. 2, A-C) at baseline and was <600 pg/mL in the majority (10/16), while the HGF levels in the two healthy donors were 60 and 1400 pg/mL. Only two patients with evaluable responses had HGF levels ≥600 pg/mL. The median HGF level in four patients with SD was 337 pg/mL (150–999), which was only slightly lower than in patients who had PD, 419 pg/mL (236–1447). There was no correlation between the basal level of HGF and response among patients who achieved SD versus those who did not (*P* = 0.783). Four of the five patients not evaluable for response, and one patient who had PD after 3 cycles, had high HGF levels, with a median value of 2891 pg/mL (577.7–11,976; Fig. 2a, c). Of the patients who had high HGF levels, only 20% (1 patient) stayed on treatment for >2 cycles, while 73% with low levels stayed on treatment for >2 cycles (Fig. 2a, b). Although there were no significant differences in the HGF levels between SD and PD groups at baseline, HGF levels decreased in three of four patients that achieved SD, while of the six evaluable patients with PD, HGF increased in four during therapy (Fig. 2a, b).

### Gene expression profiling

Of the 12 CD138^+^ samples available, 9 provided RNA suitable for GEP. The obtained data were analyzed by gene set enrichment analysis (GSEA) run between the high (*n* = 2) versus low HGF (*n* = 7) groups. Because the sample number was low in the high HGF group, a *t* test could not be conducted, and the two high HGF samples available were from inevaluable patients. To identify a gene set that was enriched in the high versus low HGF group, we selected gene sets with a false discovery rate of <0.05. This analysis revealed that interferon response genes were associated with the high HGF phenotype (Supplementary Table 1). One differentially expressed gene was Interferon regulatory factor (IRF) 4, which is required for production of immunoglobulin-secreting plasma cells [17]. In our GEP analysis, samples from patients who had high HGF had higher average IRF4 expression compared to samples from patients with low HGF (Supplementary Fig. 1). Moreover, two patients who had high HGF levels were on the trial for <2 cycles due to PD and were not evaluable for response.

## Discussion

The c-MET receptor tyrosine kinase proto-oncogene regulates cell growth, survival, and migration, and c-MET signaling after engagement of its ligand, HGF, is a contributor to the pathogenesis of myeloma. HGF and c-MET expression at the mRNA and protein levels has been found in most myeloma cell lines and primary samples [18, 19]. Studies correlating HGF levels with clinical parameters showed that HGF was elevated at diagnosis in the serum and marrows of patients [20–22] and that HGF levels were elevated in patients with more aggressive disease [21–23], correlating with an inferior prognosis [22, 24, 25]. Conversely, declining HGF levels are seen in patients responding to anti-myeloma therapies [26–28], and patients with low HGF levels were more likely to achieve high-quality responses [26, 29]. Furthermore, reduction of *MET* using shRNAs [30] or a ribozyme [31] resulted in growth inhibition and apoptosis of myeloma cell lines. Collectively, these findings provided a rationale for studying tivantinib, a small-molecule, non-ATP-competitive c-MET inhibitor which binds to and stabilizes inactive c-MET [32].

Tivantinib was well-tolerated in our patients, and the toxicities were mild and manageable, with a low rate of grade 3–4 events consistent with the known AE profile of this agent. This conclusion is supported by data collected using the MDASI, which did not indicate that therapy was poorly tolerated, with only few patients indicating >10 symptoms affecting their activity and the enjoyment of life negatively on treatment. Quality of life measurements using the EORTC QLQ-C30 and QLQ-MY20 were similarly indicative of relatively good tolerance, with overall health and quality of life judged as great by the majority.

Stable disease was seen in 4/11 evaluable patients as the best response, yielding a disease control rate of 36%, but no patients had a PR or better. A recent study by Lendvai et al. [33] evaluated the activity of cabozantinib, a multi-targeted tyrosine kinase inhibitor that suppresses MET activation. Among 12 patients, one minimal response was seen, eight patients had SD, and two suffered PD. One explanation for this lack of activity could be that HGF levels, while variable among our patients, were mostly in the low range and were not reported by Lendvai and colleagues. Of note, clinical standards for HGF levels have not been established, and we therefore based our cutoff for high vs. low based on data from the literature and from the data we obtained by examining the HGF levels in the two healthy donors (Fig. 2c). Data from Verstovsek et al. determined the HGF level in normal plasma samples to be 164–522.3 pg/mL, while the data from Sakon et al. determined the HGF level in normal plasma samples to be 90 pg/mL. Our analysis of HGF levels in two healthy donors yielded levels of 60 and 1400 pg/mL. Therefore, we conservatively set the high HGF level to be any value >2000 pg/mL, and low to be ≤2000 pg/mL [34, 35]. If we accept these definitions, this suggests the possibility that the myeloma cells from our patients were not especially dependent on HGF/c-MET signaling and that selected patients with high HGF levels could have fared better. While speculative, this hypothesis is supported by the observation that HGF levels generally tended to decrease for patients with SD compared to those who progressed. Furthermore, two patients who had high HGF levels and high IRF4 expression were on the trial for only 1 cycle each due to PD, suggesting a worse disease biology. However, median HGF levels between the two groups did not differ statistically, and the small sample size limits the ability to make strong conclusions.

After completion of our patient enrollment, several groups published data questioning the role of tivantinib as a specific MET inhibitor. Basilico et al. noted that tivantinib exerted cytotoxic activity that was not influenced by c-MET gene copy number [36]. Katayama et al. found that tivantinib showed similar potency against c-MET-addicted and nonaddicted cells [37] and that tivantinib induced a G_2_/M arrest, while other c-MET inhibitors induced *G*_0_/*G*_1_ arrest. Finally, in silico studies identified microtubules as potential tivantinib targets, and microtubule disruption was noted in treated cell lines. This led Katayama and colleagues to conclude that tivantinib inhibited microtubule polymerization in addition to inhibiting c-MET. Perhaps because the growth fraction of human myeloma cells is typically low, microtubule-targeting agents have not shown strong activity in this disease. If tivantinib does indeed work in part as a microtubule inhibitor, this could also explain its lack of efficacy in our patients.

Given the questions surrounding tivantinib, this study and the trial of Lendvai et al. with cabozantinib have not eliminated the possibility that targeting c-MET may be a useful treatment strategy for some relapsed and/or refractory myeloma patients. It would be rational to consider using more specific inhibitors in patients with high HGF levels or, better yet, with c-MET pathway activation judged by GEP or phospho-c-MET levels. Further, combination strategies with other anti-myeloma therapeutics, including proteasome inhibitors, may be an attractive option, since c-MET activation may be associated with clinical drug resistance [38].

## Electronic supplementary material

Supplementary Table 1 (DOCX 218 kb)

Supplementary Figure 1 IRF4 mRNA levels (log_2_ expression) in samples with low versus high HGF levels. Log_2_ expression levels obtained from the GEP data set. (PPTX 45 kb)
