# Supplementary material for: Phase II study of the c-MET inhibitor tivantinib (ARQ 197) in patients with relapsed or relapsed/refractory multiple myeloma
Source: Ann Hematol. 2017 Mar 23;96(6):977–85. doi: 10.1007/s00277-017-2980-3 (PMC5406425; doi:10.1007/s00277-017-2980-3)
Supplement: Supplementary file 1 — (DOCX 218 kb) [file 277_2017_2980_MOESM1_ESM.docx]

**Supplementary Table**

**Supplementary Table 1.** Genes Enriched in the High HGF (n=2) versus Low HGF (n=7) Groups with a False Discovery Rate of <0.05^1^

**
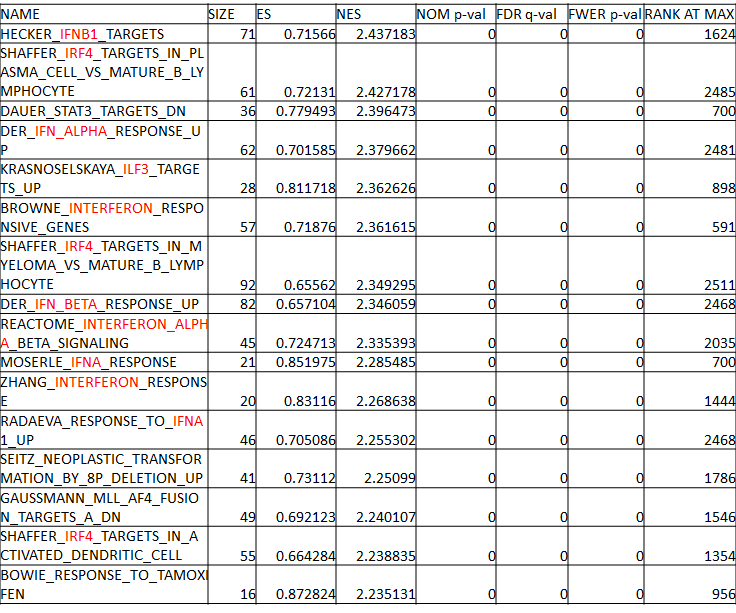
**

^1^Abbreviations used are – NAME: Name of the gene set, SIZE: Number of genes in the gene set ES: Enrichment score, NES: Normalized enrichment score, NOM p-value: Nominal p-value, FDR q-value: False discovery rate, FWER p-value: Familywise-error rate, RANK AT MAX: position rank in the maximum enrichment score list. Please see GSEA User Guide for details http://software.broadinstitute.org/gsea/doc/GSEAUserGuide (Ref: Subramanian, Tamayo, et al. (2005, PNAS 102, 15545-15550) and Mootha, Lindgren, et al. (2003, Nat Genet 34, 267-273)).
