# Supplementary figures and images for: Phase II study of the c-MET inhibitor tivantinib (ARQ 197) in patients with relapsed or relapsed/refractory multiple myeloma
Source: Ann Hematol. 2017 Mar 23;96(6):977–85. doi: 10.1007/s00277-017-2980-3 (PMC5406425; doi:10.1007/s00277-017-2980-3)

## Slide 1
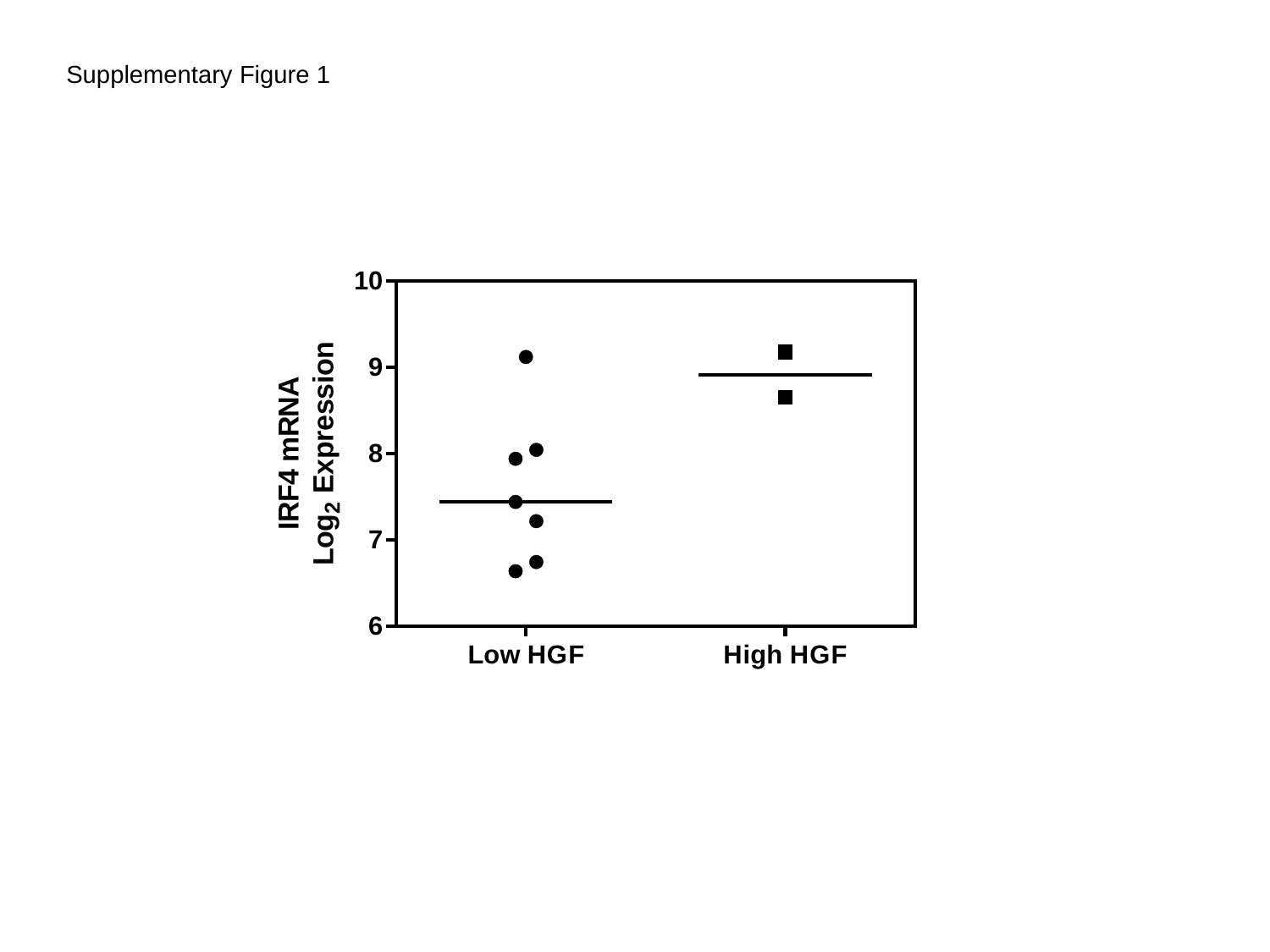

Supplementary Figure 1

Supplement: Supplementary file 2 — IRF4 mRNA levels (log2 expression) in samples with low versus high HGF levels. Log2 expression levels obtained from the GEP data set. (PPTX 45 kb) [file 277_2017_2980_MOESM2_ESM.pptx]
